# Using AI-ECG to Stratify Long-Term Mortality Risk and Prognosis in TAVR Patients

**DOI:** 10.1016/j.jacasi.2026.05.034

**Published:** 2026-08-06

**Authors:** Wence Shi, Peirou Yan, Qifeng Zhu, Guo Pu, Xianbao Liu, Cheng Li, Moyang Wang, Guannan Niu, Zhenyan Zhao, Zheng Zhou, Dejing Feng, Yongjian Wu, Jian'an Wang, Hongliang Zhang

**Affiliations:** a Department of Cardiology, Fuwai Hospital, National Center for Cardiovascular Disease, Chinese Academy of Medical Science and Peking Union Medical College, Beijing, China; b State Key Laboratory of Experimental Hematology, National Clinical Research Center for Blood Diseases, Institute of Hematology and Blood Diseases Hospital, Chinese Academy of Medical Sciences and Peking Union Medical College, Tianjin, China; c Department of Cardiology, The Second Affiliated Hospital, School of Medicine, Zhejiang University, Hangzhou, China; d State Key Laboratory of Transvascular Implantation Devices, Hangzhou, China; e Heart Regeneration and Repair Key Laboratory of Zhejiang Province, Hangzhou, China; f Transvascular Implantation Devices Research Institute, Hangzhou, China; g Peking University, Beijing, China

**Keywords:** artificial intelligence–enhanced electrocardiogram, transcatheter aortic valve replacement

## Abstract

**Background:**

Long-term mortality remains unsatisfactorily high after transcatheter aortic valve replacement (TAVR). Conventional risk models are limited in capturing subclinical electrophysiological alterations associated with poor prognosis, which can be identified on routine preoperative electrocardiograms.

**Objectives:**

The authors aim to develop and validate an artificial intelligence–enhanced electrocardiogram (AI-ECG) model for predicting long-term mortality in post-TAVR patients.

**Methods:**

A total of 711 patients with severe aortic stenosis undergoing TAVR were enrolled from 2 centers. Patients from one center were divided into training and internal validation sets (7:3), and participants from another center served as the external validation cohort. Preoperative electrocardiogram images were analyzed using a Residual Network-18 model to generate mortality risk stratification. The primary endpoint was 3-year all-cause death.

**Results:**

The AI-ECG model demonstrated comparable discrimination between the internal and external patient cohorts, with areas under the receiver operating characteristics curve of 0.767 (95% CI: 0.657-0.877) vs 0.712 (95% CI: 0.627-0.795) (*P* for DeLong test = 0.428). High-risk patients (15.5% [39 of 251]) exhibited a 61.5% (24 of 39, 95% CI: 42.8%-74.1%) 3-year mortality rate vs 16.5% (35 of 212, 95% CI: 11.4%-21.4%) in low-risk patients (84.5% [212 of 251]) (log-rank *P* < 0.001). Adjusted for comorbidities, high-risk classification independently predicted mortality (adjusted HR: 3.49; 95% CI: 1.96-6.22). Subgroup analysis did not reveal significant interaction effects of the AI-ECG model across different patient populations. Decision curve analysis confirmed clinical net benefit across threshold probabilities (0.05-0.60).

**Conclusions:**

The AI-ECG model provides noninvasive and accurate long-term risk stratification for TAVR patients, with promising clinical application value for individualized follow-up management.

Aortic stenosis (AS) constitutes a major global health burden, with severe symptomatic cases exhibiting a dismal prognosis when untreated.^1^^,^^2^ Transcatheter aortic valve replacement (TAVR) has revolutionized the management of high-risk patients, demonstrating comparable or superior outcomes to surgical alternatives in randomized trials.^3^^,^^4^ As indications expand to younger and lower-risk populations,^5^^,^^6^ optimizing long-term survival through precision risk stratification has emerged as a critical unmet need in post-TAVR care.

Current risk assessment tools, including the Society of Thoracic Surgeons (STS) score,^7^ EuroSCORE II,^8^ and J-TVT Risk Model,^9^ predominantly rely on preoperative clinical parameters. However, these models exhibit limited discriminative capacity for late mortality, failing to capture the preexisting, prognostically significant electrophysiological substrate that is reflected on the preoperative electrocardiogram (ECG) and portends long-term risk. This substrate, often subclinical, represents a risk attribute that is not quantified by static clinical scores. Concurrently, the 12-lead ECG remains an underutilized prognostic resource despite its universal availability and ability to reflect integrated cardiac pathophysiology through waveform morphology.^10^^,^^11^ Recent advances in artificial intelligence (AI) have unlocked ECG's latent predictive potential, with deep learning models demonstrating unprecedented accuracy in detecting structural heart disease, predicting incident heart failure, and identifying occult atrial fibrillation.^12^

In this context, we hypothesize that AI-enabled ECG analysis could serve as a transformative tool for post-TAVR risk stratification. Building on preliminary evidence linking ECG abnormalities to adverse outcomes in valvular heart disease,^13^ we developed a convolutional neural network–based algorithm to decode mortality-associated electrographic signatures. Our study represents the first systematic investigation into the capability of artificial intelligence–enhanced electrocardiogram (AI-ECG) to predict long-term all-cause mortality following TAVR. By leveraging the capacity of deep learning to process high-dimensional graphical data, this approach transcends conventional feature engineering limitations, potentially identifying novel prognostic biomarkers invisible to human interpretation.

## Methods

### Study population

This was a dual-center, retrospective cohort study. The inclusion criteria were as follows: 1) meeting the indications for TAVR as outlined in the *Guidelines for the Management of Patients with Valvular Heart Disease* Disease;^14^^,^^15^ and 2) availability of a standard preoperative 12-lead ECG with image quality adequate for analysis. The exclusion criteria were as follows: 1) survival of less than 1 week after TAVR; 2) acquisition of the preoperative ECG more than 2 weeks before the TAVR procedure; 3) incomplete ECG records or poor-quality scanned images; and 4) prior implantation of a permanent pacemaker or implantable cardioverter-defibrillator.

Based on these criteria, 460 consecutive patients who underwent TAVR at Fuwai Hospital between September 2012 and December 2021 were enrolled. These patients were randomly allocated in a 7:3 ratio to a training cohort and an internal validation cohort via stratified randomization. Stratification variables included age, sex, STS score, and left ventricular ejection fraction (LVEF) to ensure balanced baseline characteristics between the groups. Concurrently, 251 patients who underwent TAVR at the Second Affiliated Hospital of Zhejiang University School of Medicine between January 2016 and July 2022 and met the same criteria were enrolled as an independent external testing cohort. The AI-ECG algorithm, developed using the training cohort, was subsequently applied to both the internal testing and external validation cohorts. Based on the preoperative ECG analysis, patients in these cohorts were stratified into low-risk and high-risk groups (Figure 1).

**Figure 1:**
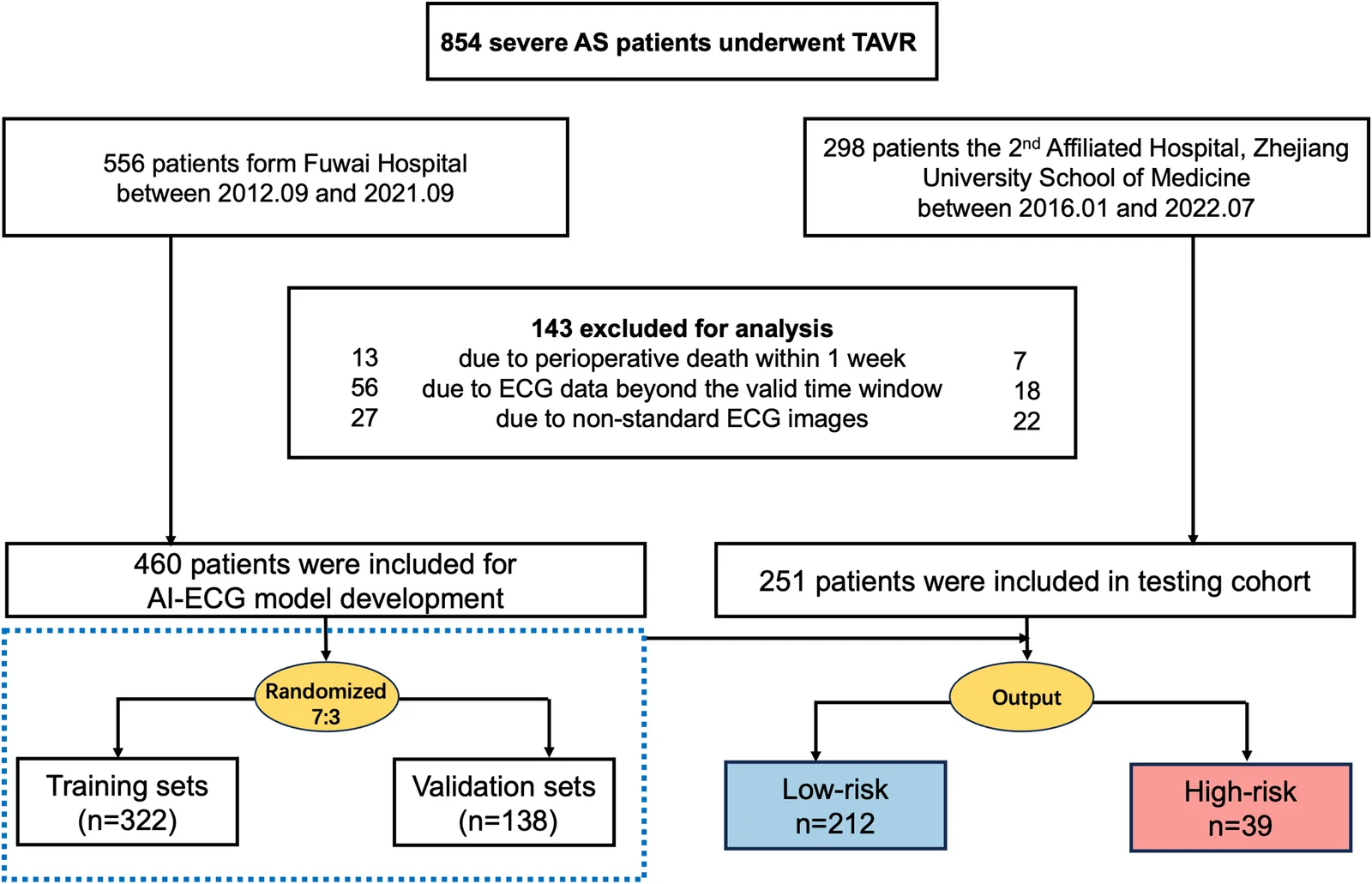
Study Flow Chart This study established an AI-ECG model to predict post-TAVR mortality. Patients were divided into internal validation (7:3) and external validation cohorts, balanced for age, sex, and left ventricular ejection fraction. The validation cohort was stratified into low-risk and high-risk subgroups by model-predicted mortality risk. AI-ECG = artificial intelligence electrocardiography; AS = aortic stenosis; ECG = electrocardiogram; TAVR = transcatheter aortic valve replacement.

This study was approved by the Institutional Review Board of Fuwai Hospital, NCCD (Approval No. 2023-2088). All patients provided written informed consent, and data confidentiality was strictly maintained throughout the study.

### Overview of AI-ECG assessment for mortality grading

In this study, we developed a deep-learning framework based on transfer learning to predict long-term mortality risk in patients after TAVR from high-resolution ECG images. First, raw ECG images were preprocessed by normalizing pixel values to the [0,1] range, cropping the borders to eliminate scan artifacts, and automatically removing watermarks via a non–black-pixel filtering technique. All images were then uniformly resized to 1,024 × 1,024 pixels to preserve critical waveform details—such as μV-level ST-segment deviations, and QT-interval variations within 40 ms.

For the model architecture, we employed an ImageNet-pretrained Residual Network-18 backbone. Its residual-block design (spanning 4 stages with a total depth of 18 layers) mitigates the vanishing-gradient problem inherent in very deep networks. To capture both local anomalies and global rhythm features, we fused multiscale pooling outputs: maximum pooling for localized irregularities and average pooling for overall rhythm characteristics. To tailor the network for binary mortality prediction, we replaced the original fully connected layer with a single-neuron output followed by a Sigmoid activation to yield a continuous probability score for death. For subsequent binary risk stratification, a cutoff value of 0.5 was used, which is a conventional threshold in binary classification tasks implying a predicted mortality risk greater than 50%. Accordingly, patients with an AI-ECG score ≥0.5 were designated as high risk, and those with a score <0.5 as low risk.

During training, we used the binary cross-entropy loss function and the Adam optimizer (initial learning rate 0.001). To retain general ECG feature representations learned in pretraining, we froze the weights of the lower convolutional layers and only fine-tuned the parameters of the last 3 layers. Model performance was evaluated on an internally held-out validation set obtained via stratified randomization (7:3 ratio) as well as on an independent external testing cohort. The primary metric was the area under the receiver operating characteristic curve (AUC), supplemented by clinically interpretable measures such as sensitivity and specificity. Compared with conventional approaches that input 224 × 224–pixel ECG images, our 1,024 × 1,024 high-definition processing ensures the capture of subtle waveform variations, thereby providing a robust technical foundation for detecting early electrophysiological remodeling. (The flowchart of AI-ECG Assessment for Mortality Grading can be found in Supplemental Figure 1).

Gradient-weighted Class Activation Mapping (Grad-CAM) was used to extract activation maps for each input image, which were then normalized to the [0, 1] range. These activation maps were overlaid onto the corresponding original ECG images to generate both standalone heatmaps and composite overlay images. Finally, the original image, heatmap, and overlay were visualized side-by-side to illustrate the regions of interest contributing to the model's prediction.

### Study endpoints and follow-up

The primary endpoint was all-cause death during the 3-year follow-up. Clinical follow-up was conducted at 1, 3, 6, and 12 months post-TAVR, with subsequent annual follow-ups via clinic visits or telephone interviews. Events were adjudicated by research personnel blinded to AI-ECG classified results per Valve Academic Research Consortium-2 criteria,^14^ with disagreements resolved by consensus.

## Statistics

Continuous variables with normal distribution were presented as mean ± SD and compared using Student's *t*-test; non-normally distributed variables were reported as median (IQR) and analyzed using the Mann-Whitney *U* test. Categorical variables were expressed as frequencies (%) and compared via chi-square or Fisher’s exact test, as appropriate. Standardized mean differences were calculated to assess baseline balance between groups (standardized mean difference <0.1 indicating negligible imbalance).

The AI-ECG model's discriminative ability was evaluated using the AUC with 95% CIs. Sensitivity, specificity, positive predictive value, and negative predictive value were calculated at a predefined risk threshold of 0.5. For time-to-event outcomes, the concordance index (C-index) with 95% CI was further computed to assess the model's time-dependent discriminatory performance in predicting 3-year all-cause mortality. Calibration of the model was assessed to evaluate the agreement between predicted 3-year mortality probabilities and observed mortality rates, using optimism-corrected calibration curves generated by 1,000 bootstrap resamples; the mean absolute calibration error and 3-year Brier score were also calculated to quantify the overall prediction error. Decision curve analysis was performed to quantify the net clinical benefit of the AI-ECG model across a range of threshold probabilities (0.05-0.6), comparing against “treat all” and “treat none” strategies.

Time-to-event outcomes were analyzed using Kaplan-Meier curves, and between-group differences were assessed via the log-rank test. Univariable Cox proportional hazards regression was first applied to identify variables associated with all-cause mortality (retention criteria: *P* < 0.10). Significant predictors, including age, prior peripheral arterial disease, atrial fibrillation, local anesthesia procedures, immediate postoperative procedural success, and AI-ECG risk stratification were incorporated into the primary multivariable model. To address potential selection bias and improve generalizability, a theory-driven Cox regression model was further constructed, which additionally incorporated clinically and pathophysiologically relevant covariates regardless of univariate statistical significance, including male sex, current/former smoking status, diabetes, hypertension, and hyperlipidemia. Adjusted HR (aHRs) with 95% CIs were calculated in both multivariable frameworks. The proportional hazards assumption was verified using Schoenfeld residuals.

Prespecified subgroup analyses were conducted across age (≤75 vs >75 years), sex, comorbidities (diabetes, atrial fibrillation), and prior percutaneous coronary intervention (PCI) status. Interaction terms between AI-ECG risk stratification and subgroup variables were tested using likelihood ratio tests. Missing data were extremely rare (<0.5% across all variables), and Little's Missing Completely At Random test confirmed the missingness was completely random. For the small number of missing values, we used mean imputation for continuous variables and mode imputation for categorical variables. All analyses were performed using R version 4.3.1 (R Foundation). A 2-sided *P* < 0.05 defined statistical significance.

## Results

### Patient characteristic

The baseline characteristics of the patients included in the AI-ECG model development are presented in Table 1. Among the 460 patients with severe AS undergoing TAVR (mean age 75.4 ± 7.3 years; 58.5% [269 of 460] male), 42 patients (9.1%) experienced the primary endpoint event at 3-year follow-up. Compared with survivors, these patients were significantly older (77.51 ± 6.63 years vs 75.16 ± 7.29 years; *P* = 0.045), had a higher prevalence of prior stroke (23.8% [10 of 42] vs 10.8% [45 of 418]; *P* = 0.026), showed significantly elevated serum creatinine levels (99.20 [85.50,119.58] μmol/L vs 88.31 [77.00, 107.03] μmol/L; *P* = 0.005), and demonstrated larger left atrial diameters on echocardiography (44.50 ± 6.73 mm vs 41.48 ± 6.17 mm; *P* = 0.003). Other baseline variables—including surgical risk scores, valvular anatomical features, and procedure-related parameters—did not differ significantly between the 2 groups.

**Table 1:** Clinical and Procedural Characteristics

	All Patients (N = 460)	Alive at 3-Year Follow-Up (n = 418)	Death Occurred at 3-Year Follow-Up (n = 42)	*P* Value
Age, y	75.37 (7.25)	75.16 (7.29)	77.51 (6.63)	0.045
Male	269 (58.5)	240 (57.4)	29 (69.0)	0.196
BMI, kg/m^2^	23.64 (3.64)	23.73 (3.55)	22.84 (4.42)	0.136
STS score, %	3.91 [2.49, 6.57]	3.90 [2.50, 6.45]	4.14 [2.40, 6.58]	0.907
Cigarette smoking				0.569
Current smoker	43 (9.4)	37 (8.9)	6 (14.3)	
Former smoker	133 (29.0)	119 (28.5)	14 (33.3)	
Never smoked	283 (61.7)	261 (62.6)	22 (52.4)	
Diabetes	117 (25.4)	107 (25.6)	10 (23.8)	0.946
Hypertension	291 (63.3)	263 (62.9)	28 (66.7)	0.755
Hyperlipidemia	291 (63.3)	271 (64.8)	20 (47.6)	0.042
Atrial fibrillation	74 (16.1)	63 (15.1)	11 (26.2)	0.099
Coronary artery disease	202 (43.9)	180 (43.1)	22 (52.4)	0.319
Previous myocardial infarction	34 (7.5)	29 (7.0)	5 (12.2)	0.309
Previous percutaneous coronary intervention	61 (13.4)	53 (12.8)	8 (19.5)	0.342
Previous heart failure	171 (37.9)	151 (36.8)	20 (48.8)	0.182
Previous stroke	55 (12.0)	45 (10.8)	10 (23.8)	0.026
Chronic kidney disease	37 (8.0)	30 (7.2)	7 (16.7)	0.063
Peripheral arterial disease	77 (16.7)	67 (16.0)	10 (23.8)	0.284
Chronic pulmonary disease	47 (10.2)	39 (9.3)	8 (19.0)	0.086
Biochemical test				
Hb, g/L	127.83 (23.56)	128.04 (23.47)	125.61 (24.65)	0.528
Plt, 10^9^/L	192.24 (62.10)	192.48 (58.72)	189.76 (90.36)	0.789
Alb, g/L	40.80 [37.40, 43.40]	41.00 [37.60, 43.40]	38.40 [36.18, 41.40]	0.003
Cr, μmol/L	89.06 [77.00, 107.03]	88.31 [76.50, 106.45]	99.20 [85.50, 119.58]	0.005
LDL, mmol/L	2.57 (1.01)	2.57 (1.02)	2.53 (0.96)	0.831
hs-CRP	2.88 [1.79, 5.56]	2.77 [1.72, 5.43]	4.32 [2.54, 6.18]	0.043
NT-proBNP, pg/mL	1,613.50 [597.08, 4,085.00]	1,530.00 [557.80, 3,877.55]	2,816.00 [1,013.70, 6,628.50]	0.023
HbA1c, %	6.26 (0.94)	6.25 (0.94)	6.37 (0.89)	0.564
TTE				
LAD, mm	41.76 (6.28)	41.48 (6.17)	44.50 (6.73)	0.003
LVEDD, mm	51.40 (8.26)	51.41 (8.16)	51.29 (9.31)	0.925
LVEF, %	55.65 (13.48)	55.76 (13.47)	54.58 (13.71)	0.589
Peak velocity, m/s	4.60 [4.20, 5.10]	4.60 [4.20, 5.10]	4.55 [4.20, 5.18]	0.728
Mean pressure gradient, mm Hg	56.50 (17.48)	56.60 (17.17)	55.55 (20.44)	0.71
Moderate or severe MR	104 (24.9)	94 (24.9)	10 (25.0)	1.000
Moderate or severe TR	53 (13.1)	49 (13.4)	4 (10.3)	0.758
Baseline CT variables				
Annular area, mm^2^	459.71 (102.05)	457.35 (100.85)	483.25 (112.02)	0.126
Bicuspid aortic valve	140 (32.1)	129 (32.6)	11 (27.5)	0.761
Aortic valve calcium score, AU	448.10 [232.00, 764.60]	434.00 [235.02, 732.82]	555.40 [214.00, 787.50]	0.430
Procedural data				
Local anesthesia	278 (62.9)	251 (62.1)	27 (71.1)	0.231
Transfemoral access	431 (98.0)	393 (98.0)	38 (97.4)	0.561
Pre-dilatation	407 (92.1)	372 (92.1)	35 (92.1)	1.000
Post-dilatation	73 (16.6)	64 (16.0)	9 (23.7)	0.320
Valve type				0.853
Self-expanded valve	425 (92.4)	387 (92.6)	38 (90.5)	
Balloon-expanded valve	35 (7.6)	31 (7.4)	4 (9.5)	
Valve size, mm				0.082
<24	103 (22.4)	96 (23.0)	7 (16.7)	
24-29	327 (71.1)	299 (71.5)	28 (66.7)	
>29	12 (2.6)	9 (2.2)	3 (7.1)	
Radiation dose, mGy	1087.00 [711.50, 1,746.00]	1,087.00 [700.00, 1,753.00]	1,121.50 [740.00, 1,667.25]	0.942
Fluoroscopy time min	20.00 [14.72, 25.25]	20.00 [14.65, 26.35]	18.00 [14.80, 20.00]	0.193
Complications after TAVR				
Device failure	17 (3.8)	14 (3.4)	3 (7.9)	0.348
Valve-in-valve implantation	55 (12.5)	50 (12.5)	5 (12.8)	1.000
Moderate or severe paravalvular leak	16 (3.7)	14 (3.5)	2 (5.3)	0.832
Moderate or severe central aortic regurgitation	2 (0.5)	1 (0.3)	1 (2.7)	0.064

Values are n (%) unless otherwise indicated. Continous variables are presented as mean (SD) or median [interquartile range].

Alb = Albumin; BMI = body mass index; Cr = Creatinine; CT = computed tomography; Hb = Hemoglobin; HbA1c = Hemoglobin A1c; hs-CRP = high-sensitivity C-reactive protein; LAD = left atrial diameter; LDL = low-density lipoprotein; LVEDD = left ventricular end-diastolic diameter; MR = mitral regurgitation; NT-proBNP = N-terminal pro–B-type natriuretic peptide; PCI = percutaneous coronary intervention; Plt = Platelet; STS = Society of Thoracic Surgeons Score; TAVR = transcatheter aortic valve replacement; TR = tricuspid regurgitation; TTE = transthoracic echocardiography.

Furthermore, baseline characteristics were well balanced between the training and internal validation cohorts (Supplemental Table 1). In the independent external testing cohort, 251 patients with severe AS undergoing TAVR (mean age 77.1 ± 7.7 years; 61.8% [155 of 251] male) were stratified by AI-ECG into a low-risk group (n = 212 of 251, 84.5%) and a high-risk group (n = 39 of 251, 15.5%). We observed notable differences in baseline profiles between this independent external cohort and the cohort used for model development (Supplemental Table 2). The external cohort was significantly older (*P* = 0.003), had a lower proportion of current or former smokers (22.7% [57 of 251] vs 38.3% [176 of 460]; *P* < 0.001), and a markedly lower prevalence of hyperlipidemia (17.1% [43 of 251] vs 63.3% [291 of 460]; *P* < 0.001). Regarding comorbidities, the external cohort showed higher rates of atrial fibrillation (24.3% [61 of 251] vs 16.1% [74 of 460]; *P* = 0.010) and chronic pulmonary disease (39.0% [98 of 251] vs 10.2% [47 of 460]; *P* < 0.001), but lower rates of prior myocardial infarction (2.4% [6 of 251] vs 7.5% [34 of 460]; *P* < 0.001), and stroke history (6.8% [17 of 251] vs 12.0% [55 of 460]; *P* = 0.039). In terms of procedural characteristics, local anesthesia (84.0% [210 of 251] vs 63.0% [279 of 460]; *P* < 0.001) and post-dilatation (53.4% [134 of 251] vs 16.6% [73 of 460]; *P* < 0.001) were more frequently used, whereas transfemoral access was less common (63.3% [159 of 251] vs 98.0% [432 of 460]; *P* < 0.001). The rate of device failure was also higher in the external cohort (17.5% [44 of 251] vs 3.8% [17 of 251]; *P* < 0.001).

### AI-ECG model performance

We observed that the AI-ECG model demonstrated comparable discriminative performance in both the internal validation cohort and the external testing cohort, achieving similar AUCs of 0.767 (95% CI: 0.657-0.877) vs 0.712 (95% CI: 0.627-0.795) (*P* for DeLong test = 0.428) (Figure 2A). The model also exhibited good time-dependent discriminatory ability for long-term mortality, with a C-index of 0.746 (95% CI: 0.682-0.809).

**Figure 2:**
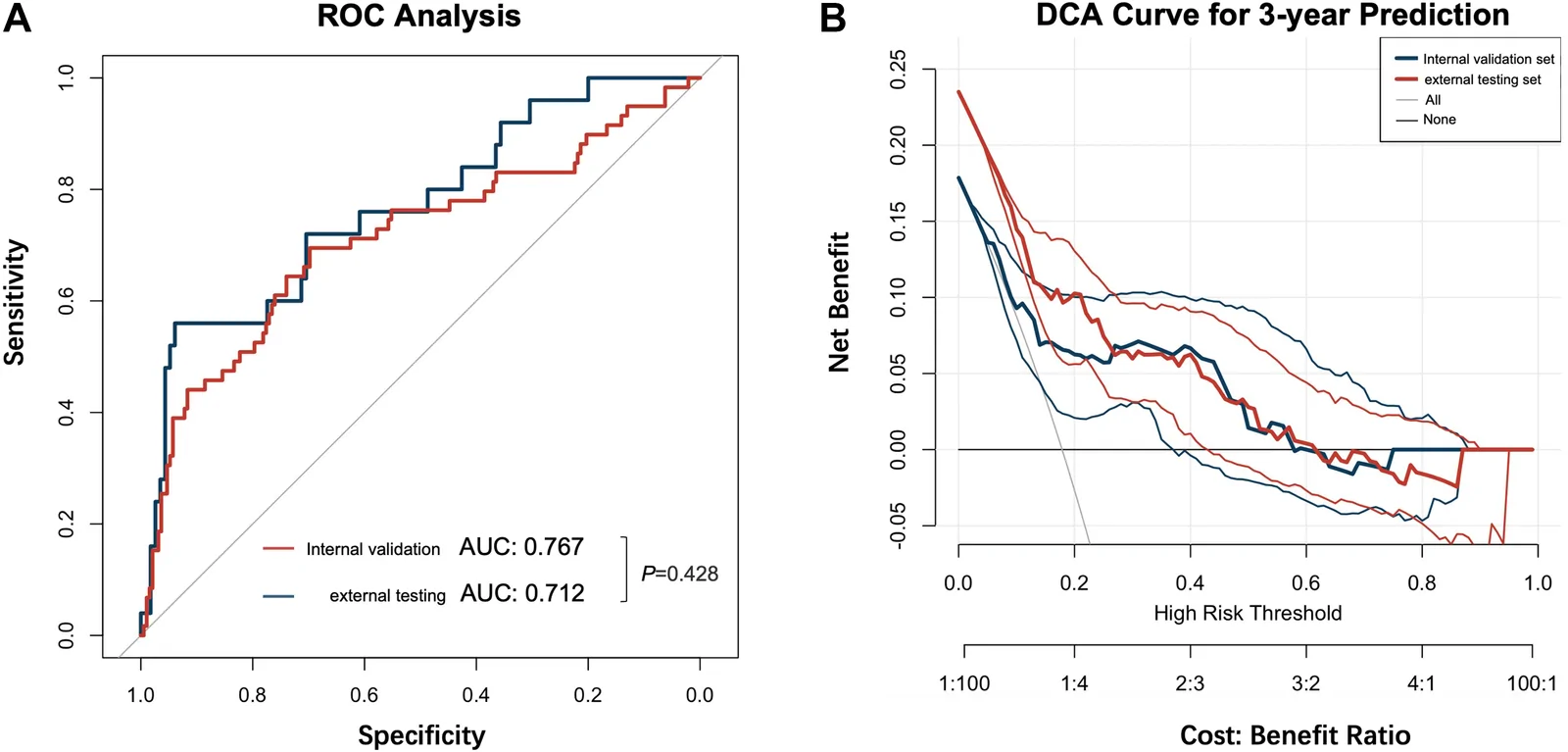
ROC and DCA The AI-ECG model showed stable discriminative ability in internal validation (AUC = 0.767) and external testing (AUC = 0.712), with no significant difference per DeLong test (*P* = 0.428, A). DCA (B) confirmed the model yielded higher net clinical benefit across threshold probabilities of 0.05 to 0.60, supporting its clinical decision-making value for transcatheter aortic valve replacement patients. AUC = area under the receiver operating characteristic curve; DCA = decision-curve analysis; ROC = receiver operating characteristic curve; other abbreviation as in Figure 1.

In the independent external testing cohort, using a risk-probability threshold of 0.5 to dichotomize predictions, 39 patients (15.5%) were classified as high risk for mortality and 212 patients (84.5%) as low risk. At this cutoff, the model's sensitivity was 69.5% (95% CI: 56.1%-80.8%), specificity was 69.8% (95% CI: 62.8%-76.2%), positive predictive value was 41.4% (95% CI: 31.6%-51.8%), and negative predictive value was 88.2% (95% CI: 81.9%-92.8%).

Calibration analysis with 1,000 bootstrap resampling was conducted to assess the agreement between predicted and observed 3-year mortality. The optimism-corrected calibration curve demonstrated satisfactory overall calibration, with predicted risk probabilities closely matching the actual mortality rates across most risk ranges. The 3-year Brier score was 0.133, indicating favorable predictive accuracy and low overall prediction error (Supplemental Figure 2).

Decision-curve analysis showed that the AI-ECG–based risk classification provided greater net clinical benefit than either “treat-all” or “treat-none” strategies over a wide range of threshold probabilities (0.05-0.60) both in the internal validation cohort and external testing cohort, indicating its potential utility for guiding clinical decision-making in TAVR patients (Figure 2B).

### AI-ECG risk stratification and prognosis

The overall all-cause mortality occurred within 3 years in 59 (Kaplan-Meier estimated rate 23.5%) of 251 patients. Kaplan-Meier analysis revealed that the cumulative mortality rate was significantly higher in the high-risk group than in the low-risk group (61.5% [24 of 39], 95% CI: 42.8%-74.1% vs 16.5% [35 of 212], 95% CI: 11.4%-21.4%; log-rank *P* < 0.001) (Figure 3). After adjusting for variables that were significant in univariate analysis (age, prior peripheral arterial disease, atrial fibrillation, local anesthesia procedures, immediate postoperative procedural success) in a multivariable Cox proportional hazards model, AI-ECG high-risk classification remained independently associated with mortality (aHR = 3.49; 95% CI: 1.96-6.22; *P* < 0.001). Further theory-driven multivariable adjustment, which additionally incorporated clinically and pathophysiologically relevant covariates regardless of univariate statistical significance, yielded consistent and robust prognostic results for AI-ECG high-risk stratification (Table 2).

**Figure 3:**
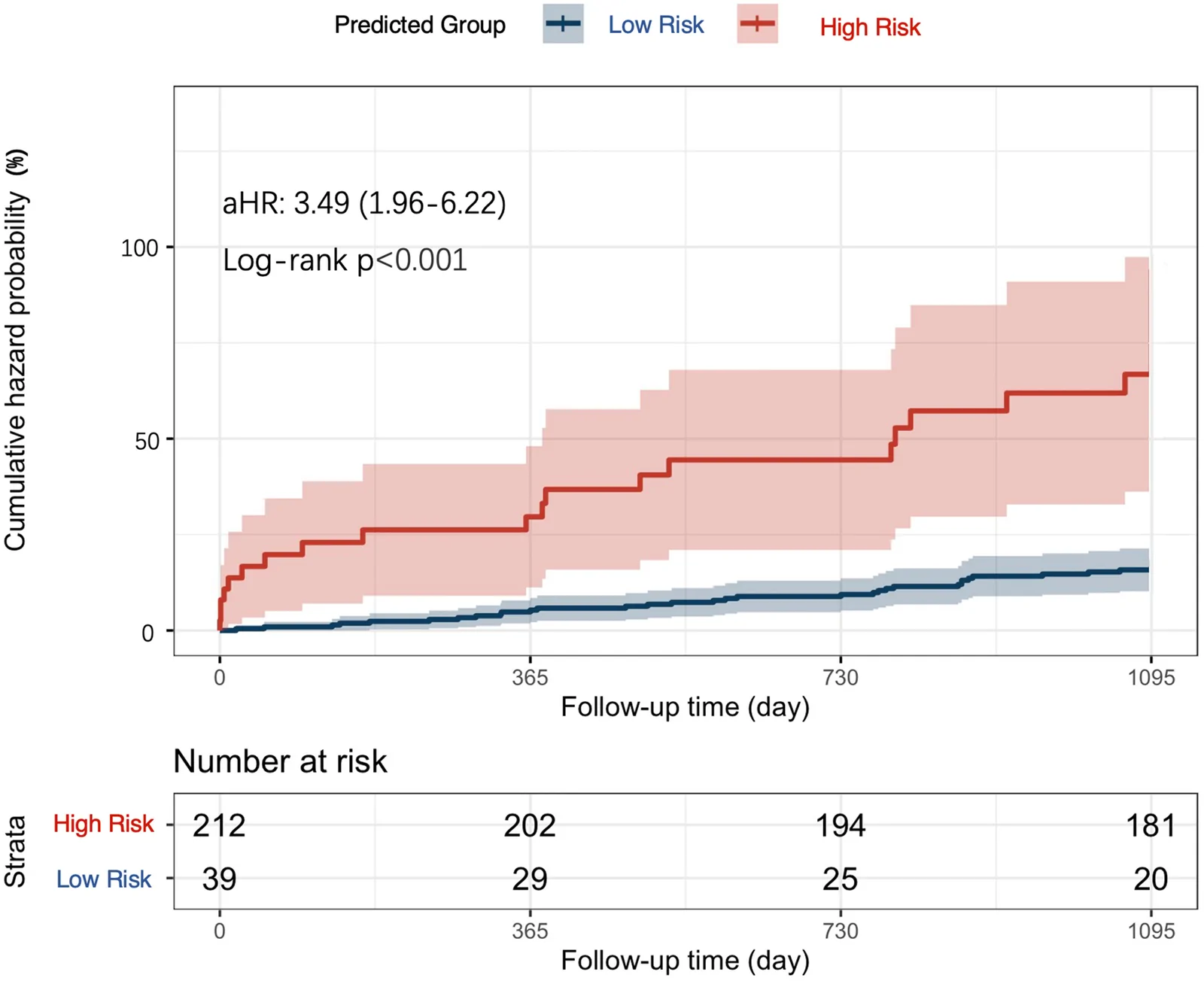
Kaplan-Meier Curves for Mortality in Patients Undergoing TAVR Kaplan-Meier analysis demonstrated significantly higher cumulative mortality in the high-risk group vs the low-risk group (61.5% vs 16.5%, log-rank *P* < 0.001). aHR = adjusted hazard ratio; TAVR = transcatheter aortic valve replacement.

**Table 2:** Multivariable Regression Analysis of the Association Between Artificial Intelligence Electrocardiography Risk Stratification and Mortality

	Model 1		Model 2		Model 3	
	HR (95% CI)	*P* Value	HR (95% CI)	*P* Value	HR (95% CI)	*P* value
Age, per 10-year increase	**1.81 (1.23-2.65)**	**0.004**	**1.59 (1.07-2.36)**	<**0.001**	**1.59 (1.08-2.35)**	<**0.001**
Male					1.84 (1.00-3.39)	0.051
Current/former smoker					0.69 (0.34-1.37)	0.287
Diabetes					0.96 (0.50-1.83)	0.892
Hypertension					1.16 (0.66-2.03)	0.605
Hyperlipidemia					1.03 (0.49-2.15)	0.940
Peripheral artery disease	1.68 (0.91-3.08)	0.093	1.13 (0.59-2.16)	0.706	1.03 (0.55-1.94)	0.919
Prior atrial fibrillation	**2.62 (1.52-4.52)**	<**0.001**	**1.82 (1.02-3.25)**	**0.042**	**1.84 (1.04-3.27)**	**0.037**
Local anesthesia procedures	**0.35 (0.20-0.62)**	<**0.001**	**0.45 (0.25-0.83)**	**0.010**	**0.42 (0.22-0.81)**	**0.009**
Procedural success	**0.43 (0.24-0.78)**	<**0.001**	**0.52 (0.28-0.95)**	**0.034**	0.56 (0.30-1.03)	0.064
High risk	**4.70 (2.72-8.13)**	<**0.001**	**3.49 (1.96-6.22)**	<**0.001**	**4.40 (2.50-7.72)**	<**0.001**

In model 1, variables exhibiting significant baseline differences between the 2 groups were included; in model 2, variables with a *P* value less than 0.01 after multivariate analysis in model 1 were included; in model 3, besides variables with significant univariate associations, additional covariates with established clinical and pathophysiological relevance to long-term mortality were further incorporated, following a theory-driven variable selection strategy. **Bold** values indicated statistically significant variables, defined as *P* < 0.05.

### Subgroup analysis

As shown in Figure 4, the association between AI-ECG high-risk classification and all-cause mortality remained robust and consistent across most predefined subgroups—including age (≤75 years vs >75 years), sex, prior PCI, atrial fibrillation, and diabetes mellitus. Multiplicative interaction terms between AI-ECG risk stratification and subgroup variables were tested using likelihood ratio tests, and all *P* for interaction >0.05. These findings demonstrate that the predictive performance of AI-ECG is consistent irrespective of underlying disease status or clinical characteristics, with no significant effect modification observed across subgroups.

**Figure 4:**
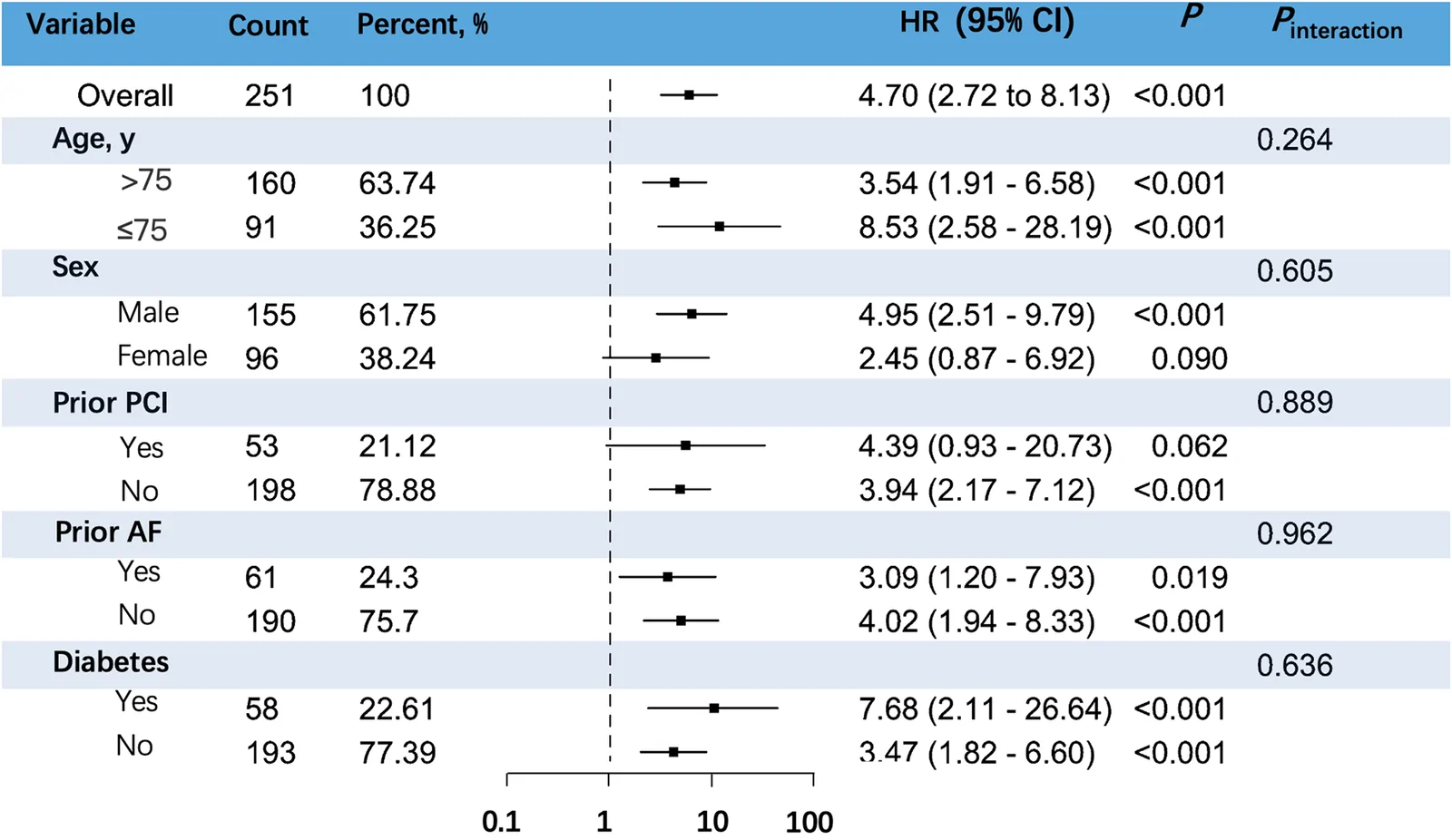
Subgroup Analysis The association between AI-ECG high-risk classification and all-cause mortality remained consistent across predefined subgroups (age ≤75 vs >75 years, sex, prior PCI, AF, diabetes mellitus). AF = atrial fibrillation; PCI = percutaneous coronary intervention; other abbreviation as in Figure 1.

### Key ECG feature interpretation

Grad-CAM visualizations revealed that, compared with low-risk patients (Figure 5A), the model's high-risk predictions were primarily driven by pronounced R-wave augmentation (Figure 5B), as well as ST-segment depression and T-wave inversion in leads V4-V6 (Figure 5C). These features may reflect ventricular hypertrophy resulting from chronic pressure overload in patients with AS.

**Figure 5:**
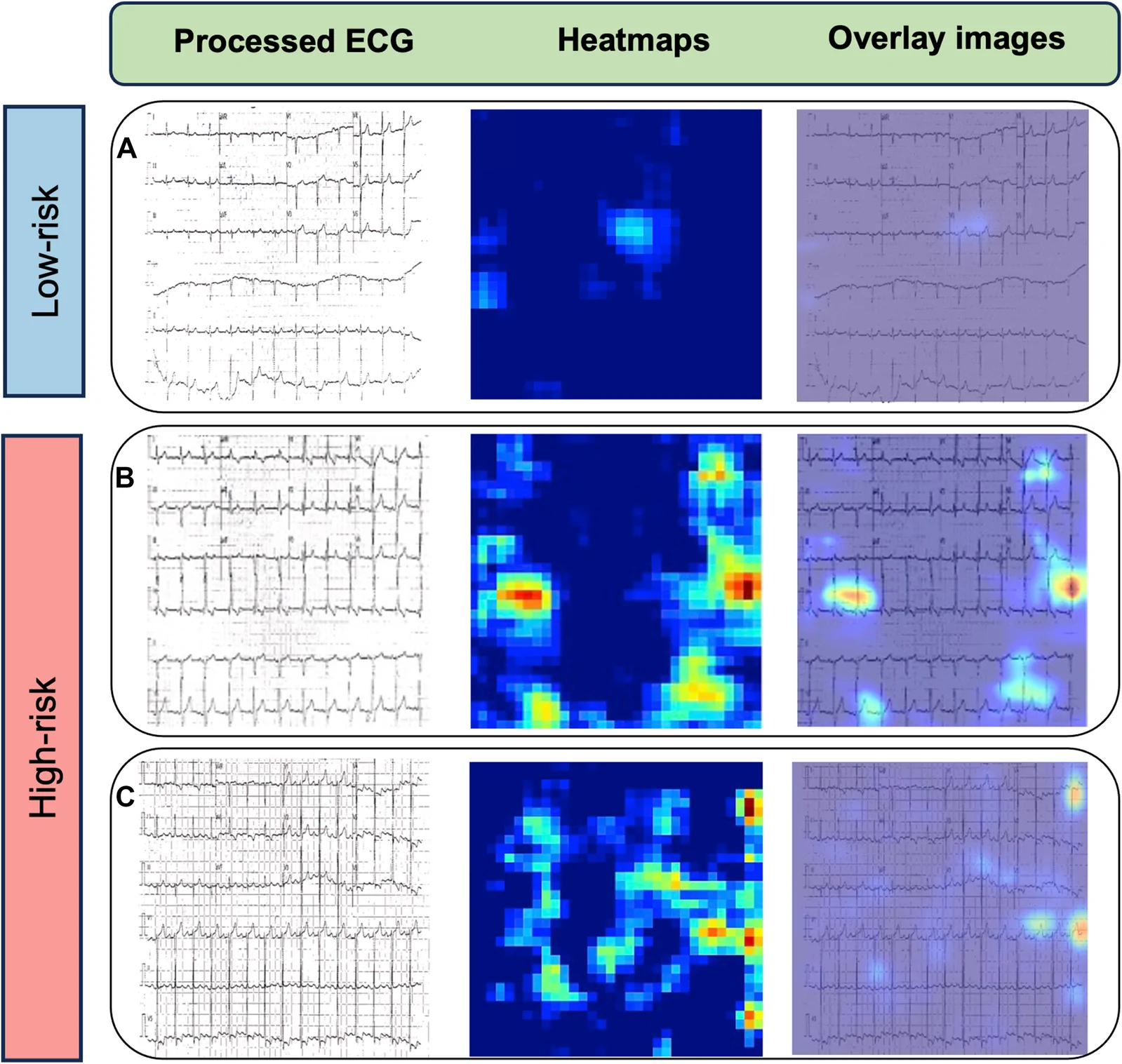
Representative ECG Examples for Low-risk and High-risk Patients Grad-CAM was used to extract and normalize (0-1) sample activation maps, which were overlaid on original ECGs to generate heatmaps and composites. (A) shows a low-risk patient's ECG; (B) and (C) show high-risk patients' ECGs. ECG = electrocardiography; Grad-CAM = Gradient-weighted Class Activation Mapping.

## Discussion

This study is the first to demonstrate that deep-learning–based ECG analysis (AI-ECG) can effectively predict long-term mortality risk in patients after TAVR. Our model showed excellent predictive performance both in an internal validation set and an independent external testing cohort, and its high-risk stratification capability significantly outperformed the conventional STS score,^16^^,^^17^ providing a novel technical paradigm for precision risk management following TAVR (Central Illustration).

**Central Illustration:**
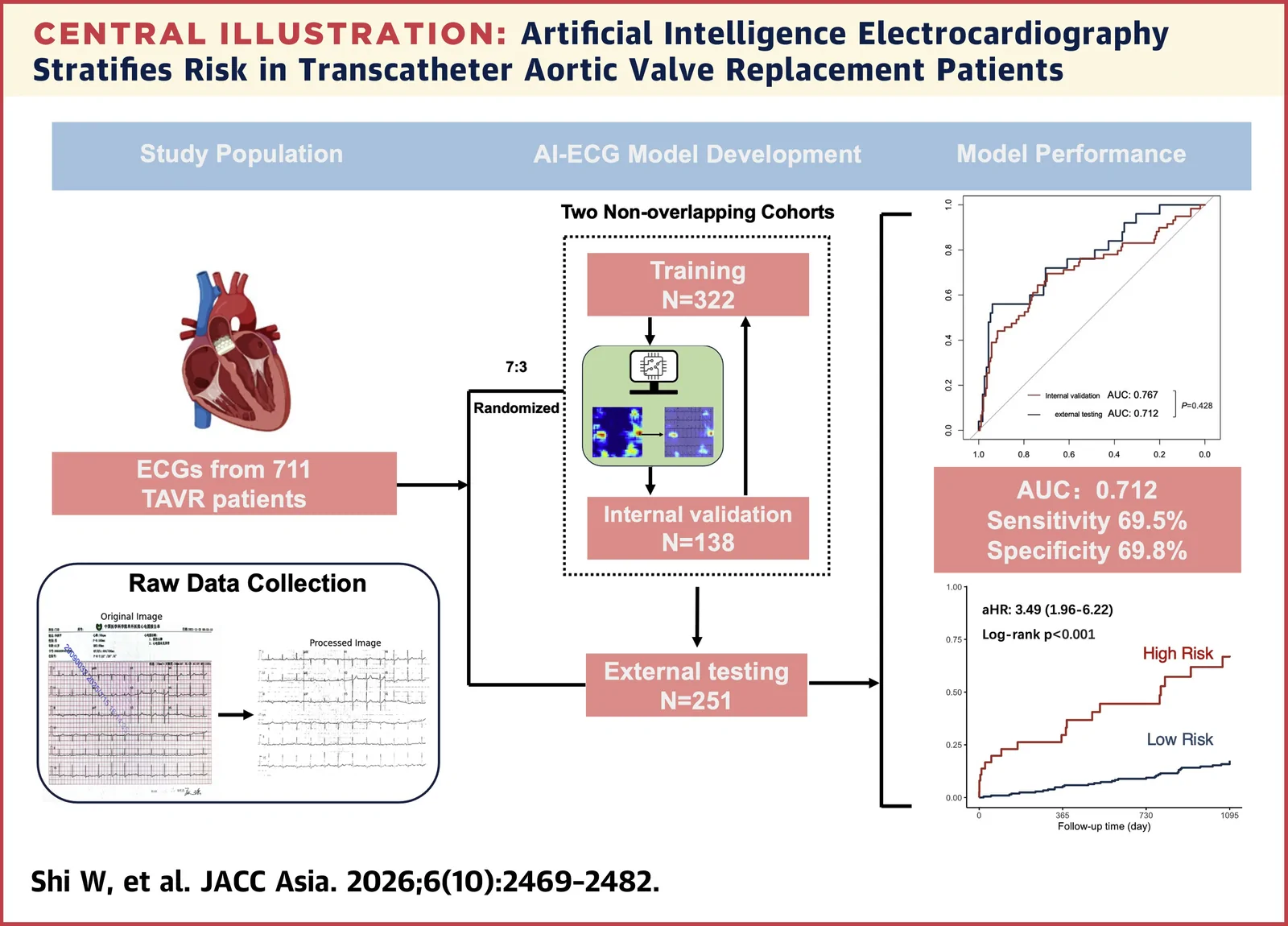
Artificial Intelligence Electrocardiography Stratifies Risk in Transcatheter Aortic Valve Replacement Patients This study included 711 TAVR patients with pre-procedural 12-lead ECG. One cohort was split (7:3) into training/internal validation; the AI-ECG model stratified the external testing cohort into low-/high-risk groups. It showed comparable discrimination (AUC 0.767 vs 0.712, DeLong *P* = 0.428) and divergent 3-year mortality (60.0% vs 16.5%, log-rank *P* < 0.001). aHR = adjusted hazard ratio; AI-ECG = artificial intelligence electrocardiography; ECG = electrocardiogram; AUC = area under the receiver operating characteristic curve; TAVR = transcatheter aortic valve replacement.

Severe AS carries a dismal natural prognosis, with a 5-year survival rate of less than 50% without intervention.^2^ TAVR, as a revolutionary therapeutic modality, has now exceeded 10,000 procedures per year in China alone, yet the 5-year post-TAVR mortality remains as high as 30% to 45%,^18^ underscoring the urgent need for robust long-term risk-stratification tools. Current clinical reliance on conventional models such as the STS score—based primarily on static preoperative variables—yields suboptimal predictive performance for late mortality (AUC 0.60-0.65)^16^^,^^17^ and fails to reflect key electrophysiological remodeling in severe AS. Although the 12-lead ECG is the most ubiquitous cardiac assessment in practice and its digitized waveforms harbor deep information on myocardial fibrosis and conduction heterogeneity,^19^ traditional ECG analysis is limited by manual measurement error and insufficient feature-extraction dimensionality, hampering capture of these latent pathological signatures.

In recent years, AI-ECG techniques—by integrating deep learning with large ECG datasets—have begun to overcome these limitations, with numerous studies demonstrating enhanced sensitivity and accuracy in detecting prognostically relevant ECG features within specific patient populations. Notably, Gal Tsaban et al^13^ developed an AI-ECG algorithm capable of grading left ventricular diastolic dysfunction in mitral regurgitation patients and stratifying their associated adverse outcomes. Furthermore, AI-ECG has proven effective in identifying clinically high-risk populations among individuals with undiagnosed structural heart disease.^20^ These findings collectively suggest that valvular heart disease–induced cardiac remodeling leaves discernible imprints on ECG patterns, which can be systematically decoded to predict clinical trajectories.

Based on these advancements, this study is the first to develop a predictive model specifically designed for the post-TAVR setting. The model was trained and internally validated using the TAVR patient cohort from Fuwai Hospital, and its ability to identify patients at high risk for long-term mortality was further confirmed through independent external validation with the patient cohort from the Second Affiliated Hospital of Zhejiang University School of Medicine. These findings lay the technological foundation for a “preoperative precision assessment–intraoperative individualized decision-making–postoperative dynamic surveillance” paradigm of comprehensive, full-cycle management in TAVR patients.

This study innovatively applies an image-based AI-ECG approach for ECG analysis, offering substantial practical advantages over traditional signal-based methods. By processing routinely stored clinical ECG scans (eg, in PDF or JPEG format), our model achieves ultra-high resolution (1,024 × 1,024) while enabling precise recognition of subtle features such as 0.1 mV–level ST-segment shifts and QT interval variations within 40 ms—enhancing spatial resolution more than 5-fold compared with conventional techniques. In an independent test cohort, the model demonstrated excellent predictive performance (AUC 0.712), and its risk-stratification capabilities offer new perspectives for optimizing health care resource allocation.

Of particular note, this technology aligns well with the current realities of health care disparity in China. On one hand, patients can upload ECG images via mobile devices to receive risk classification within 5 minutes, enabling primary care physicians to expedite referral of high-risk patients (sensitivity 69.5%) and substantially reduce decision-making time. On the other hand, most patients (84.5%) classified as low risk may safely extend follow-up intervals, with a reduction in unnecessary testing. This “low-threshold, high-accuracy” approach represents an innovative solution to the longstanding challenge of access to high-quality medical resources.

Although TAVR is an effective treatment and improves long-term cardiovascular outcomes, there remains a significant burden of late cardiovascular events following intervention that is closely associated with assessments of myocardial fibrosis and incomplete reverse left ventricular remodeling,^21^ which supports optimizing medical therapy following TAVR to improve myocardial health and patient outcome. This study demonstrates that patients identified as high risk by AI-ECG (15.5% of the external test) had an alarming 3-year mortality rate of 60.0%, underscoring the urgent need for intensified clinical management. Early initiation of heart failure therapy, such as titration of angiotensin-converting enzyme inhibitors,^22^ along with targeted antifibrotic treatment using sodium-glucose cotransporter 2 inhibitors, may offer significant benefits for this group. The recent DAPA-TAVI (Dapagliflozin After Transcatheter Aortic Valve Implantation) trial confirmed that dapagliflozin reduces the composite risk of all-cause mortality or worsening heart failure in post-TAVR patients with a history of heart failure and at least 1 additional high-risk condition (chronic kidney disease, diabetes, or left ventricular systolic dysfunction) (HR: 0.72; 95% CI: 0.55-0.95).^23^ The robust risk stratification capability of AI-ECG thus provides a valuable tool for guiding the precise application of guideline-directed medical therapy: by intensifying treatment in high-risk individuals, rehospitalization rates could be reduced, while minimizing unnecessary interventions in low-risk patients.

This study further demonstrates the broad applicability of AI-ECG risk stratification: its predictive performance remained consistent across subgroups including older adults (≥75 years), different sexes, and various comorbidities (all interaction *P* values >0.05). Notably, even in patients with cardiac rhythm disturbances, the prognostic predictive efficacy of AI-ECG remained stable. This characteristic may be related to electrophysiological mechanisms: Grad-CAM visualizations revealed that the core features of high-risk patients were primarily manifested as R-wave augmentation and characteristic ST-segment depression (>0.1 mV) in leads V4-V6. Interestingly, prior revascularization (eg, PCI) appeared to partially offset the impact of such high-risk features—subgroup analysis showed no significant interaction effect of AI-ECG risk stratification in patients with a history of PCI, suggesting that improving myocardial ischemia may confer potential prognostic benefits in patients with AS.^24^^,^^25^

In addition, the R-wave peak feature identified by AI-ECG may be associated with long-term pressure overload–induced ventricular remodeling.^26^ Cardiac magnetic resonance study^27^ has established a strong correlation between R-wave voltage and left ventricular mass index, supporting its use as a noninvasive marker of remodeling in AS. Elevated left ventricular mass independently predicts an increased cardiovascular mortality among conservatively managed patients with AS.^28^ The EARLY-TAVR (Evaluation of TAVR Compared to Surveillance for Patients With Asymptomatic Severe Aortic Stenosis) trial^29^ further underscores the benefit of early intervention, demonstrating a 50% reduction in adverse events compared with watchful waiting. Our AI-ECG model enables presymptomatic risk stratification, which highlights its potential as a practical tool for guiding timely intervention, especially in settings in which advanced imaging is limited. Prospective validation in multicenter trials remains essential to confirm these findings.

### Study limitations

First, despite the use of a standardized ECG acquisition protocol, the quality of the original ECG images remains susceptible to factors such as paper creases, low scanning resolution, and ink fading. These artifacts may introduce systematic biases into the waveform features extracted by the model. Future improvements could involve incorporating advanced image enhancement techniques, such as denoising algorithms based on Generative Adversarial Networks, to enhance the model's robustness. Second, the analysis relied solely on preoperative static ECGs, without accounting for postoperative electrophysiological dynamics such as ventricular reverse remodeling or the development of new arrhythmias. This single-time-point assessment may underestimate the prognostic impact of temporal changes in cardiac electrical activity. Integrating continuous ECG monitoring from wearable devices in future studies could enable the development of time-series prediction models with greater temporal resolution. Third, although external validation was incorporated in this study, the sample size remained limited and the dual-center population is not fully representative. Notably, substantial baseline differences existed between the training and validation cohorts, including a marked discrepancy in outcome rates (9.1% vs 23.5%), which may affect the generalizability of model performance across different clinical settings. The cohort consisted predominantly of older patients with high surgical risk profiles and severe valvular calcification, which constrains the generalizability of our findings to broader populations. As TAVR indications continue to expand toward younger and lower-risk patients, prospective multicenter studies are warranted to validate the model's performance in asymptomatic individuals and those with STS scores <4%. In addition, external validation in Western populations remains to be conducted. Moreover, some methodologic limitations warrant consideration. The event rate in the developmental cohort was substantially lower than that in the external validation cohort, which may increase the risk of model overfitting. Furthermore, the number of variables included in the multivariable Cox regression model was relatively high in relation to the number of outcome events, which also may affect the stability and generalizability of the statistical estimates.

## Conclusions

In this dual-center study, AI-ECG analysis of routine preoperative 12-lead ECG images enabled long-term mortality risk stratification after TAVR. AI-ECG–defined high-risk status was independently associated with 3-year all-cause mortality, supporting its potential clinical utility for individualized post-TAVR follow-up.

## Funding Support and Author Disclosures

This study was supported by the CAMS Innovation Fund for Medical Sciences (CIFMS) (2022-I2M-C&T-B-042, 2022-I2M-C&T-B-044, and 2022-I2M-1-023) and National High Level Hospital Clinical Research Funding (2022-GSP-QN-2). No funding was received from the industry. The authors have reported that they have no relationships relevant to the contents of this paper to disclose.
