# Supplementary material for: Using AI-ECG to Stratify Long-Term Mortality Risk and Prognosis in TAVR Patients
Source: JACC Asia. 2026 Aug 6;6(10):2469–82. doi: 10.1016/j.jacasi.2026.05.034 (PMC13700035; doi:10.1016/j.jacasi.2026.05.034)
Supplement: Supplementary file 1 [file mmc1.docx]

**Supplemental Appendix**

**Using AI-ECG to Stratify Long-Term Mortality Risk and Prognosis in TAVR Patients**

**INDEX**

Supplemental Figure 1. Flowchart of AI-ECG Assessment for Mortality Grading	2

Supplemental Figure 2. Calibration Curve of AI-ECG Model	6

Supplemental Table 1. Clinical and Procedural Characteristics between Training and Testing Set	7

Supplemental Table 2. Clinical and Procedural Characteristics between Two Non-overlapping Cohorts	10

**Supplemental Figure 1:** **Flowchart of AI-ECG Assessment for Mortality Grading**

**AI-ECG Model Development and Training Methodology**

This study implements a binary classification model based on the pre-trained ResNet18, designed to process image data and predict corresponding survival and mortality labels. The input image arrays undergo a series of preprocessing steps. First, the pixel values of the image arrays are normalized to the range [0, 1]. The images are then cropped to remove portions of the edges from the top, bottom, left, and right. Subsequently, non-black pixels are filtered out to retain only the electrocardiogram (ECG) signals. The images are resized to 1024x1024, which includes scaling and background noise removal. The dataset was randomly split into training and test sets at a 7:3 ratio for model development and validation..

**Model Architecture**

The model begins by importing the pre-trained ResNet18, which has been trained on large-scale image datasets such as ImageNet, acquiring robust image feature representation capabilities. This provides a solid foundation for subsequent fine-tuning. ResNet18 is a deep convolutional neural network composed of multiple convolutional layers, batch normalization layers, activation function layers, and pooling layers. Its core innovation lies in the introduction of residual blocks, which effectively address the issues of vanishing and exploding gradients in deep neural networks, enabling the network to be trained to greater depths.

To adapt the network for our clinical prediction task, lower layers of ResNet18 were frozen to preserve general image features, while only the final high-level layers were fine-tuned. The original fully connected layer designed for large-scale classification was replaced with a custom fully connected layer suitable for binary classification. A dropout layer with a rate of 0.5 was inserted before the final fully connected layer to reduce overfitting. The modified fully connected layer maps the features extracted by the convolutional layers to a single-dimensional output representing the probability of the sample belonging to the positive class. The Sigmoid function, defined as $\sigma\left( x \right)=\frac{1}{1+e^{-x}}$，transforms the output of the fully connected layer into a value between 0 and 1. This value represents the probability of the sample belonging to the positive class, enabling straightforward probabilistic interpretation of the model’s predictions.

During the training process, the training data is fed into the model and sequentially processed through multiple layers. Specifically, the input image first passes through a convolutional layer (conv1), which extracts local features from the image via convolution operations. Next, the data is processed by a batch normalization layer (bn1), which normalizes the input to stabilize the training process. This is followed by a ReLU activation function layer, defined *f*(*x*)=max(0,*x*), which introduces non-linearity into the network, enabling it to learn more complex features. Subsequently, the data passes through a max pooling layer (maxpool), which downsamples the feature map by taking the maximum value within local regions, thereby reducing data volume while retaining important feature information.The data then flows through four residual blocks (layer1 - layer4). The core idea of residual blocks is to introduce skip connections, allowing the network to learn residual information, expressed as *y*=*F*(*x*)+*x*, where *F*(*x*) is the residual function and *x* is the input. This structure facilitates easier training and optimization of the network. After the residual blocks, the data is processed by an average pooling layer (avgpool), which also downsamples the feature map by computing the average value within local regions. The feature map is then flattened into a one-dimensional vector for input into the fully connected layer. Finally, the output is passed through a Sigmoid layer to convert it into a probability value.During training, the binary cross-entropy loss function (BCELoss) is used to measure the difference between the model’s predictions and the true labels. The BCELoss is defined as:

$$\text{BCELoss}=-\frac{1}{N}\sum_{i=1}^{N} \left[ y_{i}\cdot\log\left( p_{i} \right)+\left( 1-y_{i} \right)\cdot\log\left( 1-p_{i} \right) \right]$$

where *yi* is the true label and *pi* is the predicted probability. The gradients of the loss function with respect to the model parameters are computed using the backpropagation algorithm, and the Adam optimizer is employed to update the model parameters. The Adam optimizer combines the advantages of Adagrad and RMSProp, adaptively adjusting the learning rate for each parameter to enhance training efficiency.

After training is completed, the model is evaluated on the test set. The output probabilities generated by the model are compared against a threshold of 0.5. Samples with probabilities greater than or equal to 0.5 are classified as positive, while those below 0.5 are classified as negative. The accuracy of the model is then calculated by comparing these predictions with the true labels.Following this, the model’s performance is further assessed using the Receiver Operating Characteristic (ROC) curve.

**Model Training Parameters**

The model was trained with a fixed 20 epochs and a batch size of 8. Early stopping was not applied, and the final model weights were saved after full training completion. All hyperparameters were set empirically based on small-sample medical image classification tasks, without systematic grid or random search.

Optimizer: Adam with a learning rate of 0.001

Loss function: Focal Loss (α=1, γ=2) was used to address the class imbalance inherent in mortality prediction

Regularization: Dropout (0.5) in the fully connected layers

Input size: 1024×1024 high-resolution ECG images

Gradients were calculated via backpropagation, and model parameters were updated using the Adam optimizer, which adaptively adjusts learning rates to improve stability and efficiency.

After training, model performance was evaluated on the held-out test set. A fixed threshold of 0.5 was applied to the predicted probabilities: patients with scores ≥ 0.5 were classified as high-risk, and those < 0.5 as low-risk. Model discrimination was assessed using accuracy and the receiver operating characteristic (ROC) curve.

**Model Strength**

The model employs a transfer learning approach, leveraging the pretrained ResNet-18 architecture to gain several advantages. Training deep networks from scratch is often resource-intensive and time-consuming; by utilizing a pretrained model, these challenges are mitigated while benefiting from its strong feature representation capabilities. The fully connected layer was modified to suit a binary classification task, making the model directly applicable to the specific clinical problem and enhancing its practicality. A Sigmoid activation function was used to convert the output into a probability value, facilitating interpretation and improving model explainability.

Using a pretrained model enabled strong feature extraction capabilities at the early stages of training, allowing the network to quickly identify key patterns, accelerate convergence, and reduce training time. The residual block architecture of ResNet-18 allows for deeper networks by addressing the vanishing and exploding gradient problems commonly encountered in traditional deep neural networks. This structure facilitates the learning of complex features and enhances overall model performance. The use of binary cross-entropy loss and the Adam optimizer further ensured training stability and efficiency—the former quantifies the discrepancy between predictions and true labels, while the latter adaptively adjusts learning rates for optimized parameter updates.

By leveraging the feature representations learned from the pretrained model, the network gains enhanced adaptability to different datasets and scenarios, thereby significantly improving its generalization capability. This allows the model to maintain stable performance when exposed to new data, without being overly sensitive to minor input variations. Moreover, the inherent universality of features learned from large-scale datasets enables the model to capture essential data patterns, reducing its reliance on the specific characteristics of the training set and effectively mitigating the risk of overfitting.

In an environment where most models use traditional 224x224 image input resolutions, our model stands out by supporting 1024x1024 high-resolution inputs, which is a significant innovation. Low-resolution inputs are akin to observing through a blurry lens, capturing only rough outlines and limited feature information. In contrast, high-resolution inputs function like using a high-definition telescope, allowing the model to capture more detailed and rich image information.

From a data perspective, a 1024x1024 resolution contains approximately 20 times the number of pixels compared to 224x224, providing the model with a vast amount of raw data for analysis and learning. In ECG classification tasks, the accuracy of classification heavily relies on waveform details. Every subtle variation in the waveform, such as the shape, amplitude, and duration of the P wave, QRS complex, and T wave, may contain crucial information about cardiac health. For example, early myocardial infarction may only manifest as minor ST-segment changes on the ECG.

Traditional low-resolution inputs often blur or lose these key details, making it difficult for the model to learn the ECG features comprehensively and accurately. With high-resolution input, our model can clearly capture these subtle changes, using them as critical features for in-depth learning and analysis. This allows the model to more accurately differentiate between various types of ECGs, significantly improving classification accuracy, enhancing diagnostic reliability, and providing stronger support for clinical decision-making.

**Supplemental Figure 2. Calibration Curve of AI-ECG Model**

Calibration analysis for 3-year all-cause mortality was performed using 1000-bootstrap resampling. As shown in the calibration curve, the optimism-corrected predicted probabilities were closely aligned with the actually observed mortality rates across most risk ranges, indicating an overall good calibration of the AI-ECG model. Minor underestimation of risk was only observed among patients with the highest predicted mortality risk. The mean absolute calibration error was 0.155.

(Black solid line: observed 3‑year mortality; Black dashed line: ideal reference line for perfect calibration (predicted probability = observed probability); Blue solid line: optimism‑corrected calibration curve based on 1000 bootstrap resamples; Gray shaded area: 95% bootstrap confidence interval; Note: 1000 bootstrap replications were performed, with a mean absolute calibration error of 0.155.)

**Supplemental Table 1: Clinical and Procedural Characteristics** **between Training and Testing Set.**

| **Variable** | **All patients**  **(n=460)** | **Training set**  **(n=322)** | **Testing set**  **(n=138)** | ***P* value** |
| --- | --- | --- | --- | --- |
| age (y) | 75.37 (7.25) | 75.15 (7.47) | 75.89 (6.71) | 0.323 |
| Male, n (%) | 269 (58.5) | 183 ( 56.8) | 86 ( 62.3) | 0.322 |
| BMI (kg/m2) | 23.64 (3.64) | 23.74 (3.42) | 23.42 (4.13) | 0.404 |
| STS score, (%) | 4.87 (3.30) | 4.84 (3.27) | 4.94 (3.36) | 0.775 |
| Cigarette smoking, n (%) |  |  |  | 0.936 |
| Current smoker | 43 (9.4) | 31 (  9.7) | 12 (  8.7) |  |
| Former smoker | 133 (29.0) | 92 ( 28.7) | 41 ( 29.7) |  |
| Never smoked | 283 (61.7) | 198 ( 61.7) | 85 ( 61.6) |  |
| Diabetes, n (%) | 117 (25.4) | 86 ( 26.7) | 31 ( 22.5) | 0.400 |
| Hypertension, n (%) | 291 (63.3) | 203 ( 63.0) | 88 ( 63.8) | 0.966 |
| Hyperlipidemia, n (%) | 291 (63.3) | 209 ( 64.9) | 82 ( 59.4) | 0.311 |
| Atrial fibrillation, n (%) | 74 (16.1) | 56 ( 17.4) | 18 ( 13.0) | 0.306 |
| Coronary artery disease, n (%) | 202 (43.9) | 153 ( 47.5) | 49 ( 35.5) | 0.023 |
| Previous myocardial infarction, n (%) | 34 ( 7.5) | 25 (  7.9) | 9 (  6.6) | 0.034 |
| Previous percutaneous coronary intervention, n (%) | 61 (13.4) | 50 ( 15.8) | 11 (  8.0) | 0.021 |
| Previous heart failure, n (%) | 171 (37.9) | 126 ( 40.3) | 45 ( 32.6) | 0.151 |
| Previous stroke, n (%) | 55 (12.0) | 43 ( 13.4) | 12 (  8.7) | 0.210 |
| Chronic kidney disease, n (%) | 37 ( 8.0) | 28 (  8.7) | 9 (  6.5) | 0.549 |
| Peripheral arterial disease, n (%) | 77 (16.7) | 56 ( 17.4) | 21 ( 15.2) | 0.663 |
| Chronic pulmonary disease, n (%) | 47 (10.2) | 29 (  9.0) | 18 ( 13.0) | 0.253 |
| **Biochemical test** | | | | |
| Hb (g/L) | 127.83 (23.56) | 127.13 (20.88) | 129.47 (28.96) | 0.333 |
| Plt (10⁹/L) | 192.24 (62.10) | 192.56 (60.40) | 191.47 (66.18) | 0.865 |
| Alb (g/L) | 49.80 (189.85) | 41.19 (6.84) | 69.83 (346.12) | 0.139 |
| Cr (µmol/L) | 97.94 (44.27) | 99.29 (49.51) | 94.79 (28.49) | 0.318 |
| LDL (mmol/L) | 2.57 (1.01) | 2.58 (1.07) | 2.54 (0.87) | 0.732 |
| CRP | 7.44 (15.22) | 7.81 (16.94) | 6.59 (10.18) | 0.496 |
| NT-pro-BNP (pg/ml) | 3720.01 (5824.81) | 3738.03 (5914.27) | 3676.69 (5627.24) | 0.922 |
| HbA1c, % | 6.26 (0.94) | 3738.03 (5914.27) | 3676.69 (5627.24) | 0.922 |
| **TTE** | | | | |
| LAD (mm) | 41.76 (6.28) | 42.06 (6.37) | 41.07 (6.04) | 0.126 |
| LVEDD (mm) | 51.40 (8.26) | 51.68 (8.54) | 50.76 (7.56) | 0.277 |
| LVEF, % | 55.65 (13.48) | 56.00 (13.35) | 54.84 (13.79) | 0.397 |
| Peak Velocity (m/s) | 4.77 (2.28) | 4.81 (2.68) | 4.68 (0.74) | 0.589 |
| Mean pressure gradient (mmHg) | 56.50 (17.48) | 56.37 (16.59) | 56.81 (19.42) | 0.807 |
| Moderate or severe MR, n (%) | 104 (24.9) | 76 ( 26.3) | 28 ( 21.7) | 0.378 |
| Moderate or severe TR, n (%) | 53 (13.1) | 34 ( 12.1) | 19 ( 15.4) | 0.449 |
| **Baseline CT variables** | | | | |
| Annular Area (mm2) | 459.71 (102.05) | 454.27 (99.12) | 472.47 (107.93) | 0.088 |
| Bicuspid aortic valve, n (%) | 140 (32.1) | 94 ( 30.9) | 46 ( 34.8) | 0.591 |
| Aortic valve calcium score, (AU) | 559.19 (452.74) | 547.12 (444.48) | 586.58 (471.72) | 0.429 |
| **Procedural data** | | | | |
| Local anesthesia, n (%) | 278 (62.9) | 197 ( 63.8) | 81 ( 60.9) | 0.814 |
| Transfemoral access, n (%) | 431 (98.0) | 304 ( 98.1) | 127 ( 97.7) | 0.599 |
| Pre-dilatation, n (%) | 407 (92.1) | 290 ( 93.5) | 117 ( 88.6) | 0.119 |
| Post-dilatation, n (%) | 73 (16.6) | 50 ( 16.3) | 23 ( 17.4) | 0.878 |
| Valve type: |  |  |  | 0.301 |
| Self-expanded valve, n (%) | 425 (92.4) | 296 ( 91.9) | 129 ( 93.5) |  |
| Balloon-expanded valve, n (%) | 35 ( 7.6) | 26 (  8.1) | 9 (  6.5) |  |
| valve size, n (%) |  |  |  | 0.246 |
| <24mm | 103 (22.4) | 75 (23.3) | 28 (20.3) |  |
| 24-29mm | 327 (71.1) | 230 (71.4) | 97 (70.3) |  |
| >29mm | 12 (2.6) | 5 (1.6) | 7 (5.1) |  |
| Radiation dose (mGy) | 2257.05 (4649.66) | 2501.82 (5090.26) | 1736.27 (3527.90) | 0.354 |
| Fluoroscopy time (min) | 29.41 (92.88) | 25.13 (50.39) | 38.45 (146.89) | 0.342 |
| Complications after TAVR | | | | |
| Device failure | 17 ( 3.8) | 11 (  3.5) | 6 (  4.5) | 0.806 |
| Valve-in-valve implantation | 55 (12.5) | 39 ( 12.6) | 16 ( 12.3) | 1.000 |
| Moderate or severe paravalvular leak | 16 ( 3.7) | 13 ( 4.2) | 3 ( 2.3) | 0.590 |
| Moderate or severe central aortic regurgitation | 2 ( 0.5) | 1 ( 0.3) | 1 ( 0.8) | 0.607 |

BMI= Body Mass Index; STS= Society of Thoracic Surgeons Score; Hb= Hemoglobin; Plt= Platelet; Alb= Albumin; Cr= Creatinine; LDL= Low-Density Lipoprotein; CRP=C-reactive protein; HbA1c= Hemoglobin A1c; TTE= Transthoracic Echocardiography; LAD= left atrial diameter; LVEDD= Left Ventricular End-Diastolic Diameter; MR= Mitral Regurgitation; TR=Tricuspid Regurgitation; PCI= Percutaneous Coronary Intervention

**Supplemental Table 2:** **Clinical and Procedural Characteristics between** **Two Non-overlapping Cohorts**

| **Variable** | **All patients**  **(n=711)** | **Fuwai Hospital**  **(n=460)** | **2^nd^ Affiliated Hospital, Zhejiang University School of Medicine**  **(n=251)** | ***P* value** |
| --- | --- | --- | --- | --- |
| age (y) | 75.99 (7.45) | 75.37 (7.25) | 77.12 (7.70) | 0.003 |
| Male, n (%) | 424 (59.6) | 269 (58.5) | 155 (61.8) | 0.441 |
| Cigarette smoking, n (%) |  |  |  | <0.001 |
| Current/former smoker | 233 (32.8) | 176 (38.3) | 57 (22.7) |  |
| Never smoked | 477 (67.2) | 283 (61.7) | 194 (77.3) |  |
| Diabetes, n (%) | 175 (24.6) | 117 (25.4) | 58 (23.1) | 0.550 |
| Insulin use, n (%) | 49 (28.0%) | 31(26.5%) | 18 (31.0%) | 0.087 |
| Hypertension, n (%) | 432 (60.8) | 291 (63.3) | 141 (56.2) | 0.077 |
| Hyperlipidemia, n (%) | 334 (47.0) | 291 (63.3) | 43 (17.1) | <0.001 |
| Atrial fibrillation, n (%) | 135 (19.0) | 74 (16.1) | 61 (24.3) | 0.010 |
| Previous myocardial infarction, n (%) | 40 ( 5.7) | 34 ( 7.5) | 6 ( 2.4) | <0.001 |
| Previous percutaneous coronary intervention, n (%) | 114 (16.1) | 61 (13.4) | 53 (21.1) | <0.001 |
| Previous stroke, n (%) | 72 (10.1) | 55 (12.0) | 17 ( 6.8) | 0.039 |
| Peripheral arterial disease, n (%) | 123 (17.3) | 77 (16.7) | 46 (18.3) | 0.666 |
| Chronic pulmonary disease, n (%) | 145 (20.4) | 47 (10.2) | 98 (39.0) | <0.001 |
| **Procedural data** | | | | |
| Local anesthesia, n (%) | 489 (70.6) | 279 (63.0) | 210 (84.0) | <0.001 |
| Transfemoral access, n (%) | 591 (85.4) | 432 (98.0) | 159 (63.3) | <0.001 |
| Pre-dilatation, n (%) | 651 (93.8) | 407 (91.9) | 244 (97.2) | 0.008 |
| Post-dilatation, n (%) | 207 (30.0) | 73 (16.6) | 134 (53.4) | <0.001 |
| Valve type: |  |  |  | 0.200 |
| Self-expanded valve, n (%) | 649 (91.3) | 425 (92.4) | 224 (89.2) |  |
| Balloon-expanded valve, n (%) | 62 ( 8.7) | 35 ( 7.6) | 27 (10.8) |  |
| valve size, n (%) |  |  |  | <0.001 |
| <24mm | 151 (21.8) | 103 (23.3) | 48 (19.2) |  |
| 24-29mm | 520 (75.3) | 327 (74.1) | 193 (77.2) |  |
| >29mm | 21 (3.0) | 12 (2.7) | 9 (3.6) |  |
| Complications after TAVR | | | | |
| Device failure | 61 ( 8.7) | 17 ( 3.8) | 44 (17.5) | <0.001 |
| Valve-in-valve implantation | 79 (11.4) | 55 (12.5) | 24 ( 9.6) | 0.292 |
